# RNA-Seq reveals different responses to drought in Neotropical trees from savannas and seasonally dry forests

**DOI:** 10.1186/s12870-021-03244-7

**Published:** 2021-10-12

**Authors:** Mariane B. Sobreiro, Rosane G. Collevatti, Yuri L. A. dos Santos, Ludmila F. Bandeira, Francis J. F. Lopes, Evandro Novaes

**Affiliations:** 1 Laboratório de Genética & Biodiversidade, Instituto de Ciências Biológicas, Universidade Federal de Goiás, Goiânia, GO 74690-900 Brazil; 2 Laboratório de Genética e Genômica de Plantas, Escola de Agronomia, Universidade Federal de Goiás, Goiânia, GO 74690-900 Brazil; 3 Laboratório de Fisiologia Vegetal, Instituto de Ciências Biológicas, Universidade Federal de Goiás, Goiânia, GO 74690-900 Brazil; 4 Laboratório de Genética Molecular, Departamento de Biologia, Universidade Federal de Lavras, Lavras, MG 37200-900 Brazil

**Keywords:** Bignoniaceae, Differential gene expression, Water deficit, Plasticity, *Tabebuia* Alliance, Transcription factors

## Abstract

**Background:**

Water is one of the main limiting factors for plant growth and crop productivity. Plants constantly monitor water availability and can rapidly adjust their metabolism by altering gene expression. This leads to phenotypic plasticity, which aids rapid adaptation to climate changes. Here, we address phenotypic plasticity under drought stress by analyzing differentially expressed genes (DEG) in four phylogenetically related neotropical Bignoniaceae tree species: two from savanna, *Handroanthus ochraceus* and *Tabebuia aurea,* and two from seasonally dry tropical forests (SDTF), *Handroanthus impetiginosus* and *Handroanthus serratifolius.* To the best of our knowledge, this is the first report of an RNA-Seq study comparing tree species from seasonally dry tropical forest and savanna ecosystems.

**Results:**

Using a completely randomized block design with 4 species × 2 treatments (drought and wet) × 3 blocks (24 plants) and an RNA-seq approach, we detected a higher number of DEGs between treatments for the SDTF species *H. serratifolius* (3153 up-regulated and 2821 down-regulated under drought) and *H. impetiginosus* (332 and 207), than for the savanna species. *H. ochraceus* showed the lowest number of DEGs, with only five up and nine down-regulated genes, while *T. aurea* exhibited 242 up- and 96 down-regulated genes. The number of shared DEGs among species was not related to habitat of origin or phylogenetic relationship, since both *T. aurea* and *H impetiginosus* shared a similar number of DEGs with *H. serratifolius*. All four species shared a low number of enriched gene ontology (GO) terms and, in general, exhibited different mechanisms of response to water deficit. We also found 175 down-regulated and 255 up-regulated transcription factors from several families, indicating the importance of these master regulators in drought response.

**Conclusion:**

Our findings show that phylogenetically related species may respond differently at gene expression level to drought stress. Savanna species seem to be less responsive to drought at the transcriptional level, likely due to morphological and anatomical adaptations to seasonal drought. The species with the largest geographic range and widest edaphic-climatic niche, *H. serratifolius*, was the most responsive, exhibiting the highest number of DEG and up- and down-regulated transcription factors (TF).

**Supplementary Information:**

The online version contains supplementary material available at 10.1186/s12870-021-03244-7.

## Background

Water is generally the main limiting growth factor of plants in Tropical regions [1, 2]. Plants constantly monitor water availability and temperature to adjust their metabolism accordingly. These metabolic adjustments are driven by gene expression changes, which cause phenotypic plasticity and may induce memory (acclimation) [3, 4] and priming [5] in transcriptional response, enhancing the ability of plants to cope with similar conditions in the future. As such, these metabolic changes can aid rapid adaptation to climate changes [6, 7], relaxing selective pressures [8], especially in environments with recurrent stresses [4]. Savannas are marked by seasonal droughts and recurrent fires [9, 10], and the study of plants from these ecosystems could reveal important molecular adaptations to drought.

Several studies have investigated transcriptional responses to drought stress in model and domesticated plants, such as *Arabidopsis thaliana* [11], *Solanum lycopersicum* [12], *Sorghum bicolor* [13], *Medicago trunculata* [14], *Oryza sativa* [15], *Populus trichocarpa* [16] and *Eucalyptus* spp. [17, 18]. Canonical mechanisms usually involve abscisic acid (ABA) and gene expression changes in ABA-related genes, jasmonic acid (JA) biosynthesis signaling pathway, sugar catabolism to produce compatible solutes, antioxidants to scavenge reactive oxygen species (ROS), stomatal closure and photosynthesis shut down [13, 19–21]. However, studies with wild plants, especially those from the Neotropics, are still scarce [22]. Wild species adapted to different environmental conditions, such as drier savannas versus more humid forests, may have evolved different molecular responses to drought. Understanding the plastic responses to water deficit in plants from different ecosystems could elucidate the evolution of important mechanisms and adaptations.

Climatic models predict a worldwide temperature increase of up to 4 °C by the mid-century [23]. Models forecast a 36 and 15% rise in the frequency and duration of meteorological drought, respectively, with a 1.5 °C increase in global temperature [24, 25]. Analyses of three decades of global climate data show that the amplitude (of affected area) and frequency of severe droughts are more variable in the Southern Hemisphere [26]. The same study also shows that the Brazilian Cerrado biome is undergoing a drying trend [26], threatening agriculture and natural biodiversity in the region [27, 28].

The Cerrado biome has savannas and seasonally dry forests and is characterized by average annual rainfall below 1600 mm and distinct rainfall seasonality [29]. Transition between savanna and seasonally dry tropical forest (SDTF) in the region is mainly due to soil fertility, with savannas restricted to dystrophic soils with high aluminum and low organic matter content, and recurrent fires [30, 31]. As a result, savannas have up to three times less above-ground biomass and are therefore more susceptible to drought [31]. The ground layer of savannas is dominated by C4 grasses, which are susceptible to recurrent fires during seasonal droughts, whereas the closed canopy of SDTFs tends to exclude grasses and fire [30, 32]. Thus, study of molecular and phenotypic plasticity of savanna species exposed to seasonal drought may reveal genes and metabolic pathways that are important for adaptation to this abiotic stress.

In order to compare the molecular responses of savanna and forest species, we analyzed gene expression levels through RNA-Seq in four phylogenetically related Bignoniaceae tree species with contrasting life histories [33]. *Tabebuia aurea* and *Handroanthus ochraceus* are both widely distributed in Neotropical savannas, but *T. aurea* occurs both in seasonally dry and wet savannas, whereas *H. ochraceus* is only found in savannas and occasionally SDTF. *Handroanthus impetiginosus* is widely distributed in SDTFs in South and Mesoamerica, and *Handroanthus serratifolius* occurs in SDTFs as well as rain forests and riparian forests [34]. Although *Handroanthus impetiginosus* prefers hot dry climates, it is found under broad range of annual temperatures, matching the current general conditions of SDTFs. The species has no soil fertility preference, occurring in soils with a wide range of cation exchange capacity, pH and base saturation [35, 36]. *Handroanthus serratifolius* also occurs in hot climates, but with a greater variation in rainfalls and soil fertility [37], being more generalist (broader climatic and soil niche) than *H. impetiginosus*. Although *T. aurea* and *H. ochraceus* are adapted to hot dry climates, they have a narrower niche breadth for annual rainfall and temperature than the two SDTF species, occurring in areas with less than 2000 mm of rain and low temperature seasonality, in line with the current general conditions of Neotropical savannas [38, 39].

We previously sequenced the genome of the pink ipê (*H. impetiginosus*), predicting and annotating 31,688 genes [40]. Here, we used this genome to perform an RNA-Seq to analyze the gene expression levels of two savanna and two SDTF species under drought and irrigated control*.* The objective of this study was to test whether savanna and SDTF species respond differently to drought at the gene expression level. Understanding how plants adapted to different environmental conditions (e.g. savanna vs. SDTF) cope with abiotic stress may indicate their potential to respond to climate change. It could also reveal specific genes and metabolic pathways that might be important for drought tolerance.

## Results

### Differentially expressed genes in response to drought

A total of 448.8 millions of 2 × 100 nt paired-end sequences were generated for the four species. The number of reads per individual ranged from 15.52 to 38.66 million (Additional File 1: Table S1), with an average of ~ 40% GC content and Phred > 30 for most of the bases sequenced. Filtering removed ~ 1.3% of the sequences (Additional File 1: Table S1). Approximately 78% of the reads mapped uniquely, i.e. into only one gene of the reference genome with high mapping quality (MAPQ > = 30). This mapping rate ranged from 68.4 to 80.6% depending on the species and treatment (Additional File 1: Table S2). The difference in mapping rate among species was negligible, even though a single reference genome from *H. impetiginosus* [40] was used. The percentage of non-mapped reads was very low across all species (maximum of 0.02%, Additional File 1: Table S2).

**Fig. 1:**
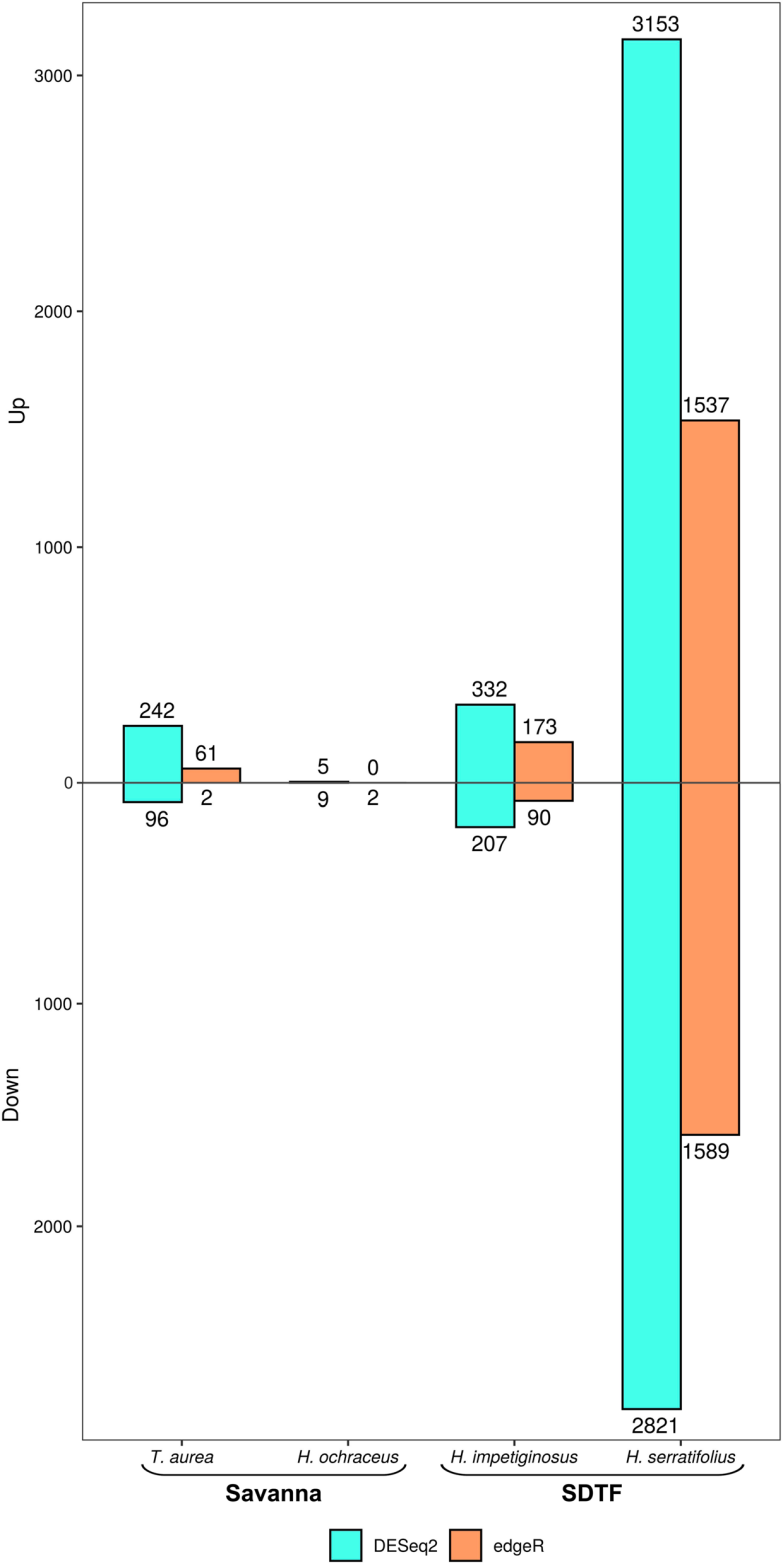
Total number of differentially expressed genes (DEGs) separated in up-regulated (above the line) and down-regulated (below the line), for the four analyzed species. The DEGs were identified using the packages DESeq2 and edgeR according to the figure legend, with a false discovery rate FDR < 0.05

DESeq2 detected more differentially expressed genes (DEGs) between treatments than edgeR (Fig. 1) for all the species. DESeq2 also detected both up- and down-regulated genes for all the species, whereas edgeR identified no up-regulated genes for *H. ochraceus* (Fig. 1). In order to comprehensively explore the molecular effects of drought, i.e. with fewer type II (false negative) errors, DEGs detected by DESeq2 were used for further analyses.

*Handroanthus serratifolius* had a higher number of DEGs between treatments (3153 up-regulated and 2821 down-regulated), followed by *H. impetiginosus* (332 and 207)*. Handroanthus ochraceus* exhibited the lowest number of DEGs, with only five up- and nine down-regulated genes (Fig. 1). In order to determine whether the fewer DEGs for *H. ochraceus* was due to the smaller number of individuals analyzed (one for drought and two for wet treatments), we randomly selected the same number of individuals for the other species and used DESeq2 to count the number of DEGs. Despite lowering the sample size for the other species, *H. ochraceus* still had a lowest number of DEGs (946 DEGs for *T. aurea*, 32 for *H. ochraceus*, 2138 for *H. impetiginosus* and 2360 for *H. serratifolius*). For a general view of the DEG, see Heatmap in Additional File 2: Fig. S1.

**Fig. 2:**
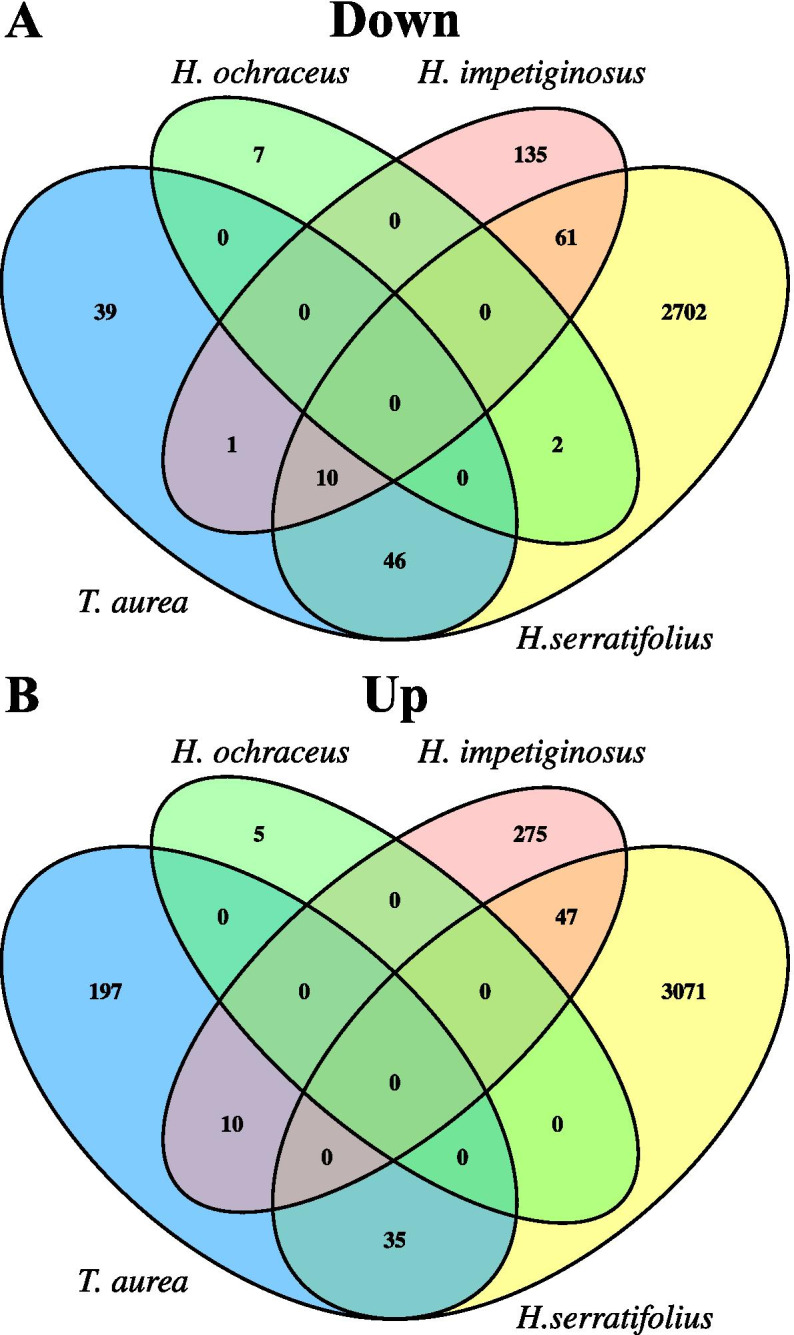
Number of differentially expressed genes (DEGs) exclusive for each species and shared among the four analyzed species, identified using the package DESeq2. **a** Venn diagram for down-regulated genes. **b** Venn diagram for up-regulated genes

**Fig. 3:**
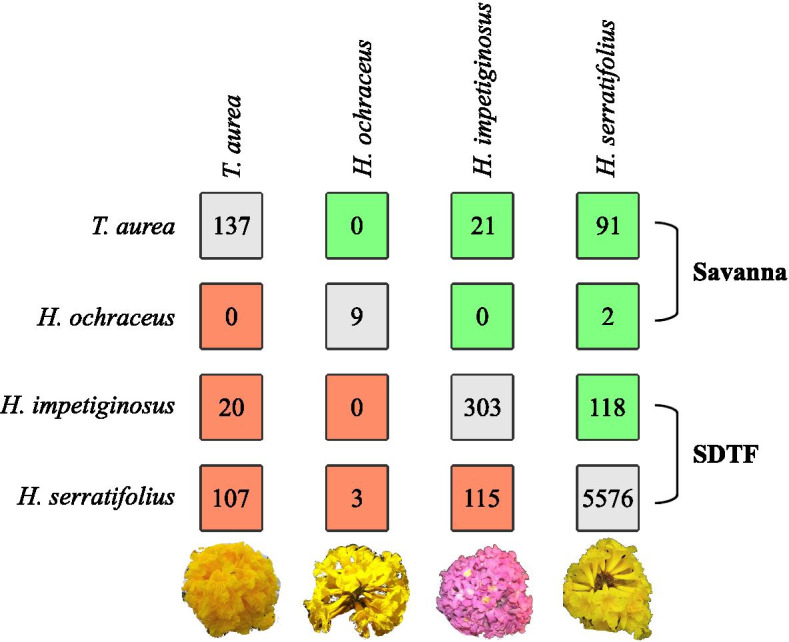
Number of conserved expressed genes (CEG - mint green boxes above diagonal), divergent expressed genes (DiEG - salmon boxes below diagonal) and exclusive expressed genes (gray boxes in the diagonal) among species. CEGs indicate differentially expressed genes (DEGs) for which the direction of expression is maintained between pairs (e.g., up-regulated in both species). The CEG are the sum of the shared down- and up-regulated genes from Fig. 2. DiEG represent DEGs with different direction of expression between species (e.g., up-regulated in one and down-regulated in another)

Species shared fewer up- than down-regulated genes (Fig. 2). Shared up- and down-regulated genes were denominated conserved expressed genes (CEGs) to differentiate them from DEGs that occurred in two species but with different directions of expression (e.g., up-regulated in one species and down-regulated in another). In the latter case, genes were referred to as diverged expressed genes (DiEGs). The number of CEG varied between species pairs (Fig. 3; Additional File 1, Table S6), but was not related to phylogenetic relationship, since both *T. aurea* and *H impetiginosus* had similar CEGs to *H. serratifolius*. Most DEGs were exclusive to one species or were diverged expressed genes (DiEGs), i.e. had a different pattern of expression between species (Fig. 3; Additional File 1: Table S6).

### Functional annotation and transcription factor analysis

The number of enriched gene ontology (GO) terms for *T. aurea, H. impetiginosus, and H. serratifolius* was 70 (Additional File 1: Table S3), 75 (Additional File 1: Table S4) and 692 (Additional File 1: Table S5), respectively. *Handroanthus ochraceus* had no enriched GO terms due to the small number of up- (five) and down-regulated (nine) genes.

**Fig. 4:**
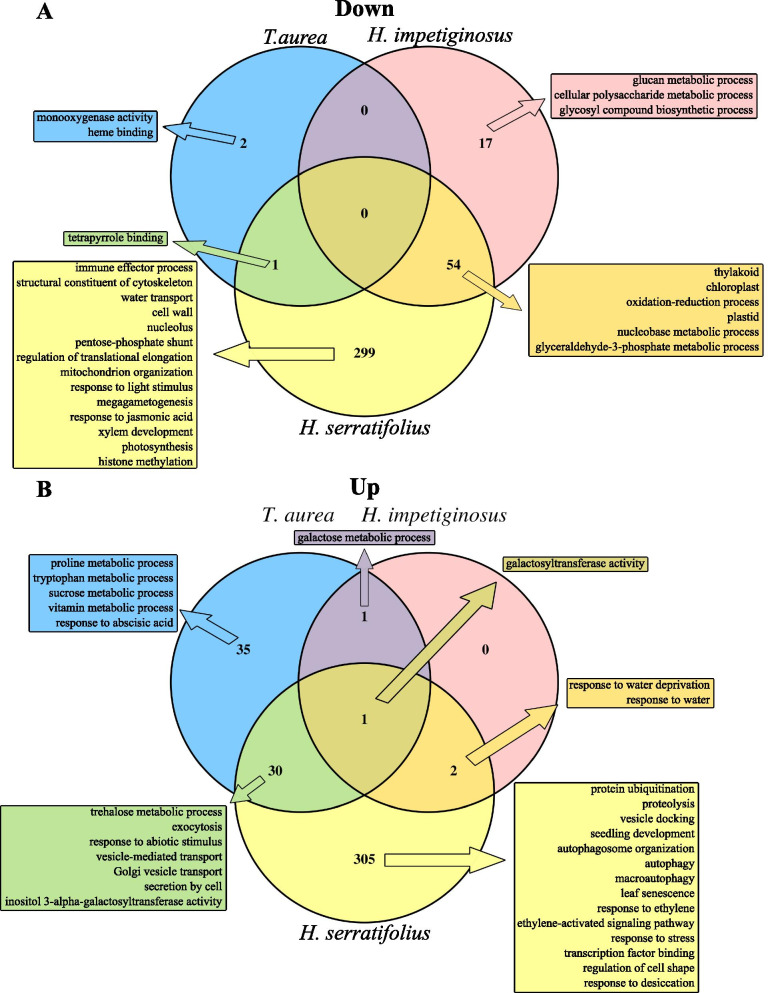
Number of enriched GO terms among differentially expressed genes (DEGs) exclusive for each species and shared among them. **a** Venn diagram of GO enriched terms among down-regulated genes. **b** Venn diagram of GO enriched terms among up-regulated genes. The side boxes depict common and unique major canonical categories enriched among up- and down-regulated genes to drought stress

**Fig. 5:**
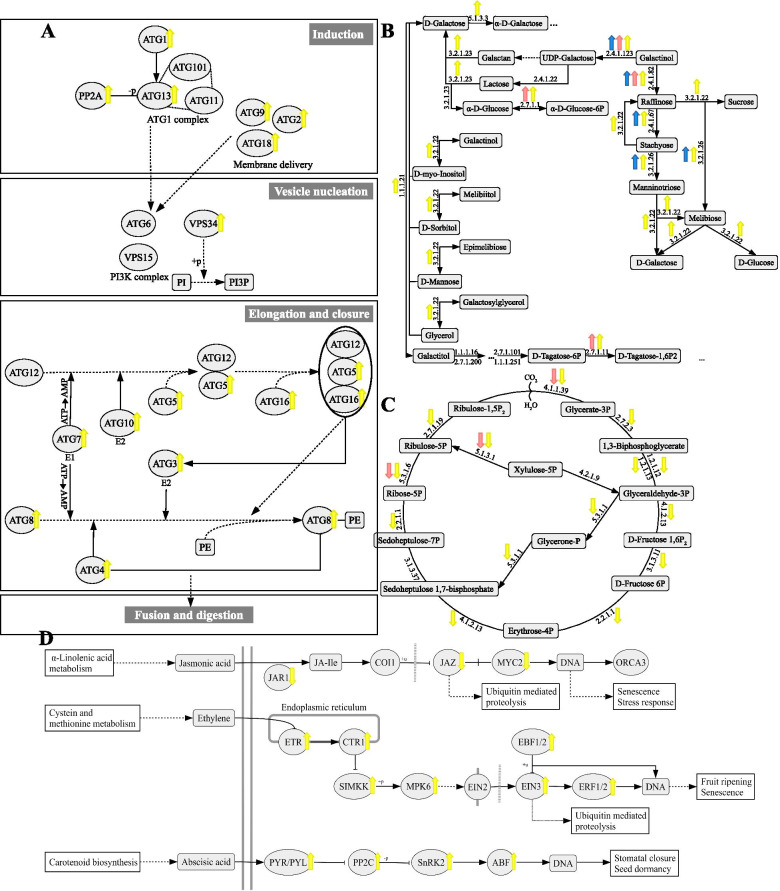
Representative pathways containing up- (thick up arrows) and down-regulated (thick down arrows) genes. Blue arrows indicate *Tabebuia aurea* genes, pink *H. impetiginosus* and yellow *H. serratifolius*. **a** Autophagy pathway with DEG in *H. serratifolius*. **b** Galactose pathway with DEG in *T. aurea*, *H. impetiginosus* and *H. serratifolius*. **c** Calvin-Benson cycle showing DEG in *H. impetiginosus* and *H. serratifolius*. **d** Hormonal and signal transduction pathways with DEG in *H. serratifolius*. Circular shapes represent proteins, while rectangular shapes represent chemical compound, DNA or other molecules; filled lines ended with arrows represent activation, molecular interaction or the direction of pathways; filled lines with perpendicular endline represent inhibition; filled lines with perpendicular middleline represent dissociation; dashed lines represent indirect link or unknown reaction; connector lines represent association; +p and -p represent phosphorylation and dephosphorylation, respectively; +u represents ubiquitination; numbers encode Enzyme Code (EC) identifiers

All four species shared a low number of enriched GO terms (Fig. 4). Of the 354 enriched GO terms among the down-regulated genes of *H. serratifolius*, 54 were shared with *H. impetiginosus* and only one with *T. aurea* (Fig. 4). Of the 338 enriched GO terms among the up-regulated genes of *H. serratifolius*, two were shared with *H. impetiginosus*, 30 with *T. aurea*, and only one among the three species (Fig. 4). Because *H. serratifolius* had many more DEG compared to the other species (Fig. 1), it presented many exclusive enriched GO terms and pathways, including exclusive DEG in the autophagy pathway (Fig. 5A). On the other hand, all three species shared an enrichment for the galactosyltransferase activity term (GO:0008378, Additional File 1: Table S6), as shown in the galactose metabolism pathway depicting the DEG in these three species (Fig. 5B). *H. impetiginosus* and *H. serratifolius* (Additional File 1: Tables S4 and S5) exhibited enrichment for water deprivation and chloroplast elements, as well as the Calvin-Benson cycle pathway (Fig. 5C). In general, the species seem to exhibit different mechanisms of response to drought stress (Fig. 4, Additional File 1: Table S6). This was confirmed by a Jaccard distance between samples, using the GO enriched categories as binary markers. All distances in the matrix were greater than 0.85 (Additional File 2: Fig. S2), close to the maximum Jaccard distance of one.

**Fig. 6:**
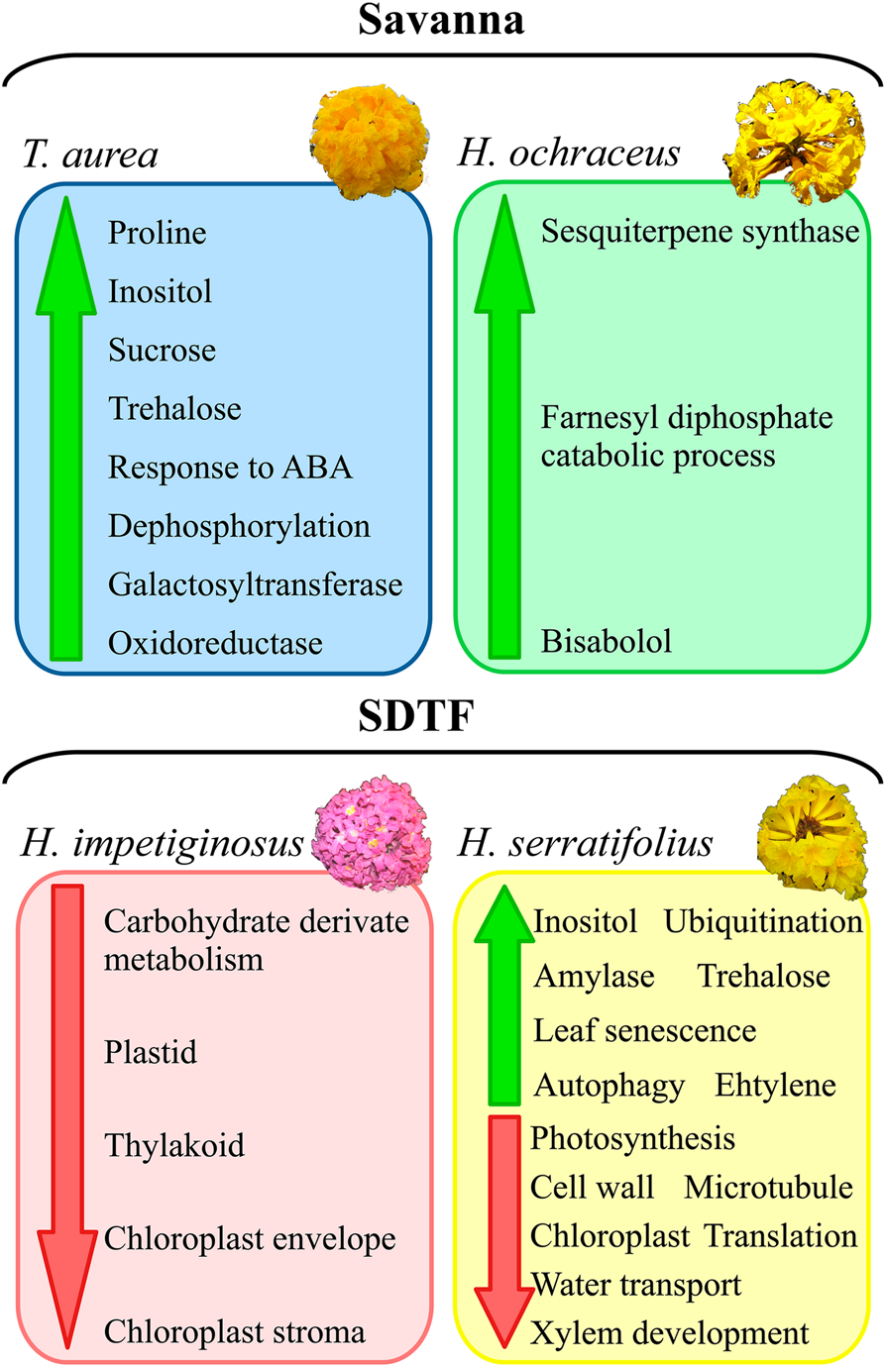
Summary of the main strategies involved in the responses to drought stress of each species, based on GO enriched terms and KEGG Orthology (KO) terms. Green up-arrow represents up-regulated genes and red down-arrow represents down-regulated genes

Although *H. ochraceus* had no enriched terms, three up-regulated genes were related to terpene synthesis. These genes were exclusively found as differentially expressed in *H. ochraceus* (Additional File 1: Table S6), indicating that this terpene-related response was specific to this savanna species. In general, *T. aurea* seems to respond to drought by accumulating compatible solutes in an ABA-mediated fashion, with pronounced dephosphorylation activity (Fig. 6, Additional File 1: Table S3 and Table S6). *H. impetiginosus* appears to reduce photosynthetic machinery activity and pathways related to the production of photosynthetic pigments in response to drought (Figs. 5C and 6, Additional File 1: Table S4 and Table S6).

On the other hand, *H. serratifolius* showed a multitude of metabolic changes (Fig. 6). The GO enriched categories indicate that *H. serratifolius* attempted all possible mechanisms. Many different categories were enriched, including those expected under canonical response to drought, such as photosynthesis shut down through stomatal closure and the activation of transcription factors (Figs. 4 and 6, Additional File 1: Table S5 and Table S6). The enriched GO categories of *H. serratifolius* demonstrate down-regulation of photosynthesis and of pentose phosphate pathways (Figs. 4A and 5B), as observed in *H. impetiginosus*. *Handroanthus serratifolius* also exhibited enrichment for terms related to compatible solute accumulation (Figs. 4B and 5B), as detected for *T. aurea*. Among down-regulated genes (Additional File 1: Table S5), enriched GO terms for water transport, xylem development, megagametogenesis, and immune effector process were observed (Fig. 4A), while in up-regulated genes, evident cell degradation signaling was triggered, as indicated by enriched GO categories for autophagosome, autophagy (Figs. 4B and 5A), mitophagy, nucleophagy, proteolysis mediated by ubiquitin and lipid catabolism processes (Additional File 1: Table S5 and Table S6). This demonstrates that *H. serratifolius* degraded its proteins and lipids to obtain energy as the photosynthetic machinery was down-regulated. *H. serratifolius* also seems to invest in premature reproduction, with enriched GO terms for ethylene signaling, leaf senescence, seedling development and seed germination among up-regulated genes (Fig. 5D, Additional File 1: Table S5 and Table S6).

**Fig. 7:**
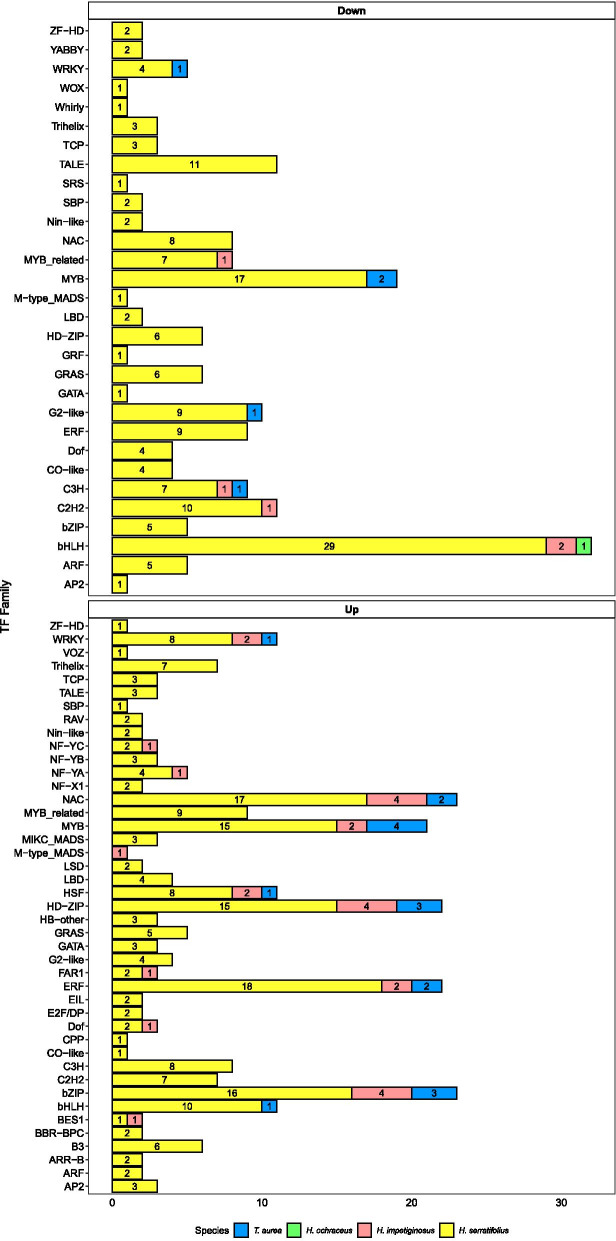
Transcription factors families and their corresponding absolute frequencies among down- and up-regulated genes for the four analyzed species. The full names for each transcription factor family (e.g. Basic helix-loop-helix for bHLH) is in Abbreviations section, as well as in Table S7

Among down-regulated genes in all the species, 175 transcription factors (TF) were found: one from *H. ochraceus*, five from *T. aurea*, five from *H. impetiginosus* and 164 from *H. serratifolius* (Fig. 7, Additional File 1: Table S6). These encompassed 30 different families: none were shared between all species; three were shared between *T. aurea* and *H. serratifolius* (*WRKY*, *G2-like* and *MYB*); one between *T. aurea*, *H. impetiginosus* and *H. serratifolius* (*C3H*); two between *H. impetiginosus* and *H. serratifolius* (*C2H2* and *MYB_related*); one between *H. impetiginosus*, *H. ochraceus* and *H. serratifolius* (*bHLH*); and 23 families were exclusive to *H. serratifolius*.

A total of 255 putative TFs were up-regulated under drought (Fig. 7, Additional File 1: Table S6) in *T. aurea* (17), *H. impetiginosus* (26) and *H. serratifolius* (212). No TFs were found among the five up-regulated genes of *H. ochraceus*. Among the up-regulated TFs, we found 43 different families: seven were shared among all three species (*bZIP*, *WRKY*, *NAC*, *HSF*, *ERF*, *HD-ZIP* e *MYB*); one between *T. aurea* and *H. serratifolius* (bHLH); five between *H. impetiginosus* and *H. serratifolius* (*NF-YA*, *BES1*, *DOF*, *FAR1* e *NF-YC*); one exclusive family in *H. impetiginosus* (*M-type MADS*); and 29 exclusive families in *H. serratifolius*.

We also assessed TFs shared between up- and down-regulated genes for each species. Two of the 10 families identified in *T. aurea*, none of the 17 in *H. impetiginosus* and 24 of the 48 in *H. serratifolius* were shared between up- and down-regulated genes. The *HSF* family was unique to up-regulated genes in all species. *BES1*, *FAR1*, *NF-YA*, and *NF-YC* were unique to up-regulated genes in *H. impetiginosus* and *H. serratifolius*.

## Discussion

A greenhouse experiment was conducted with three biological replicates to identify consistent drought-induced gene expression changes in *Tabebuia* Alliance species from savannas and seasonally dry tropical forests (SDTF). The number of differentially expressed genes (DEG) varied among species. Overall, SDTF species were more responsive to drought stress, with a higher number of DEGs than their savanna counterparts. The more generalist species, *H. serratifolius,* was the most responsive to drought stress and showed the highest number of DEGs (5974 genes with DESeq2). By contrast, *H. ochraceus,* the more specialist species (i.e. with a narrower niche, more specific to seasonal savannas), exhibited the lowest number of DEGs (only 14). In sorghum, less DEG was also observed in the drought tolerant genotype compared to the more susceptible one [13]. These results indicate that tolerant genotypes, such as the savanna trees studied here, may be less responsive to drought stress at the transcriptional level. One hypothesis for this difference is that savanna species may have a more developed root system, capable of absorbing and even storing more water [41]. Indeed, although the roots of the seedlings used in our experiment were confined in pots, the dry biomass of recovered roots was significantly higher for savanna species (3.9 g for *T. aurea* and 3.3 g for *H. ochraceus*) compared to those from SDTFs (1.4 g for *H. impetiginosus* and 1.2 g for *H. serratifolius*) (Additional File 2: Fig. S3).

Savanna trees have a highly developed root system that reaches the water table and may be subject to less drought stress [42, 43]. This morphological adaptation of savanna species roots may reduce the need for molecular adaptations to drought stress at the transcriptional level [44]. The shoots of savanna trees may also have specific adaptations to drought stress, such as better osmotic regulation (as shown here for *T. aurea*). However, both savanna and SDTF species lost shoot biomass under drought (Additional File 2: Fig. S3). On the other hand, all four species were far more resilient in terms of root biomass (Additional File 2: Fig. S3). As a result, the shoot/root biomass allocation decreased significantly under drought.

Our results also show that the molecular plasticity response to drought stress may be expressed differently in different species. Most genes and functional categories were specific to each species. The number of conserved expressed genes (CEGs) was lower compared to the number of diverged (DiEGs) and exclusive DEGs (Fig. 3). Moreover, phylogenetic relationship was not the main factor determining the number of CEGs between species. *T. aurea* shares a proportionately larger number of CEGs with *H. serratifolius* (Figs. 2 and 3) than the latter’s more closely related species *H. impetiginosus*. Habitat of origin also did not determine the number of CEGs, given that the savanna species *T. aurea* and *H. ochraceus* had no CEGs, whereas *T. aurea* shared CEGs with the SDTF species *H. serratifolius*. It is important to note that this result may have been affected by the low number of differentially expressed genes (DEG) in *H. ochraceus*. However, most DEGs in the SDTF species (*H. impetiginosus* and *H. serratifolius*) were not CEGs. For example, only 33.6% of the 539 DEGs in *H. impetiginosus* were CEGs (Figs. 2 and 3). These results indicate that most of the transcriptional responses to drought were specific to each species, at least under the shoot analysis conditions used here. Transcriptional responses in the root should also play an important role, although it was not the focus of this study. However, a previous RNA-Seq study indicated that the transcriptional responses in the shoots were the most important factor involved in drought tolerance of kabuli chickpea (*Cicer arietinum* L.), compared to the gene expression changes observed in the roots [45].

### Functional responses to drought stress

We improved the functional annotation of *H. impetiginosus* genes predicted in the reference genome by adding Gene Ontology, Enzyme codes, KEGG orthology, PFAM and PANTHER terms to the genes [40]. This annotation is provided as supplementary material (Additional File 1: Table S6) for the scientific community. Our findings show that all four species use different strategies to cope with drought. The more generalist species, *H. serratifolius*, seems to be the most sensitive to water deficit, both because of its higher number of DEGs and the several enriched GO terms among down- and up-regulated genes when compared to the other species. This extensive transcriptional remodeling indicates an extreme response to drought. Among the down-regulated genes of *H. serratifolius,* enrichment was observed for xylem development, photosynthesis, glycolysis, pentose phosphate shunt, rRNA and tRNA metabolism, ATP synthesis, immune response genes, carbohydrate and nucleotide biosynthesis. These functional categories indicate that *H. serratifolius* shuts down its primary metabolic processes due to severe stress. On the other hand, among up-regulated genes, enrichment was observed for terms related to autophagy (Fig. 5A), protein ubiquitination and proteolysis (Fig. 6). This combination may indicate that plants were undergoing senescence [46], which was confirmed by visual symptoms, such as yellowing and detachment of *H. serratifolius* leaves in the experiment. In fact, the shoot biomass of *H. serratifolius* was highly impacted by drought, resulting in a remarkable change in biomass allocation with ~ 55% reduction in the shoot/root ratio (Additional File 2: Fig. S3). Additionally, we found ethylene terms among up-regulated genes (Fig. 5D), which is related to leaf senescence and premature reproduction attempt. Although the plants were very young, premature reproduction is also evident in the presence of seed germination and seedling development enriched categories among up-regulated genes (Additional File 1: Table S5).

Compatible solute accumulation seems to be the main response of *T. aurea* to drought, evident in the enriched terms related to galactose, hexose and trehalose metabolic processes among up-regulated genes (Fig. 6 and Additional File 1: Table S3). Galactose metabolic process was also enriched among *H. impetiginosus* up-regulated genes (Fig. 5B). Compatible solutes such as galactinol can act in osmotic adjustment, membrane and macromolecule stabilization and osmoprotection. Contribution of compatible solutes to water deficit tolerance has been widely reported [47–50].

Plants under water stress tend to exhibit a decrease in photosynthesis rate. The set of enriched genes in *H. impetiginosus* and *H. serratifolius* are typical of drought-imposed risks to the photosynthetic apparatus. Photosynthetic down-regulation in response to drought involves two phases: stomatal closure and ROS accumulation initially, followed by photoinhibition [51]. In both SDTF species, the apparent down-regulation in photosynthesis under drought occurred in the second phase, since down-regulation of genes belonging to non-stomatal factors was observed, including the Calvin-Benson cycle (Fig. 5C). Both species showed mechanisms associated with down-regulation of photosynthesis-pigment synthesis and chloroplast organization. This is evident in enriched GO terms for chlorophyll biosynthetic process, chlorophyll metabolic process, pigment biosynthetic process and porphyrin-containing compound biosynthetic process among down-regulated genes. The two SDTF species also had down-regulated genes in the pentose-phosphate pathway, indicating that hexose degradation occurred preferably via glycolysis rather than the hexose monophosphate shunt pathway [52].

Moreover, *H. serratifolius, H. impetiginosus* and *T. aurea* presented enriched GO terms for galactosyltransferase activity among up-regulated genes (Figs. 4B and 5B). *Galactosyltransferase* is related to galactinol synthesis, a raffinose precursor. Both galactinol and raffinose are compatible solutes that can be used for osmosis regulation and act as antioxidants [53]. In addition to exhibiting the lowest number of DEGs, *H. ochraceus* was the only species with terpene-related terms among up-regulated genes. Increased terpenoid concentration has been reported as a tolerance mechanism for water deficit [54, 55]. As such, *H. ochraceus* was probably less sensitive to drought stress due to a combination of different strategies, such as terpenoid accumulation, investment in a large root system, leaf senescence (Additional File 2: Fig. S3) and photoassimilate accumulation [56].

Given the higher number of DEGs (5974) in *H. serratifolius*, many functionally enriched categories were specifically found in this species. Some of the enriched categories in up-regulated genes were related to stress, such as response to water deprivation, abiotic stimulus, salt stress, toxic substances, chemicals and osmotic stress (Additional File 1: Table S5). In addition to the large number of DEGs, these categories indicate that *H. serratifolius* was under severe stress.

Given this condition, *H. serratifolius* showed clear molecular responses to cope with severe drought, including down-regulation of many primary metabolic processes. In addition to photosynthetic shut down, enriched categories were observed among down-regulated genes for rRNA and ribosome biosynthesis, as well as purine and pyrimidine biosynthesis (Figs. 4, 6 and Additional File 1: Table S5). This down-regulation of primary metabolic processes suggest that *H. serratifolius* obtained energy from its own proteins and fatty acids through proteolysis and autophagy. This was evident in the enrichment of several GO categories among up-regulated genes, such as ubiquitin-dependent protein catabolism, protein ubiquitination, cellular catabolic process, fatty acid catabolic process, autophagy, mitophagy and endocytosis (Figs. 4, 6 and Additional File 1: Table S5). Enrichment was also observed for ATP hydrolysis and protein localization to vacuoles among up-regulated genes. Vacuoles play an important role in protein degradation and recycling [57].

Our findings also indicate an integrative response to drought stress, possibly through transcription factors (TFs), since several annotated TFs were differentially expressed (Fig. 7). The large number of predicted transcription factors among up- and down-regulated genes indicate that these master regulators might be important drivers of the transcriptional changes observed in the different species under drought. Transcription factors induce molecular responses to biotic and abiotic stimuli by modulating the expression of several specific genes. *bHLH* is the second largest family of transcription factors in plants and has been reported to be involved in tolerance to abiotic stresses, such as water deficit [58–61]. The *bHLH* family was present among up- and down-regulated genes, activating and repressing specific pathways. On the other hand, the *HSF* family was unique to up-regulated genes in *T. aurea*, *H. impetiginosus* and *H. serratifolius*. Overexpression of *HSF* in transgenic *Arabidopsis* lines enhances drought tolerance [62, 63]. Jiang et al. [64]⁠ noted that exposure to ABA also increased *HSF* expression in maize, indicating an ABA-dependent link between *HSF* and abiotic stresses.

The plant hormone abscisic acid plays a crucial role in the response to drought [19]. In *H. serratifolius*, many genes of the abscisic acid pathway were up-regulated in response to drought (Fig. 5D). These responses are mediated by the *ABA-responsive element* (*ABRE*) binding protein or *ABRE binding factor* (*AREB/ABF*) [65]. In the PlantTFDB database, *AREB* transcription factors are classified as *bZIP*, since they contain this protein domain. In our study, *bZIP* is one of the TF families with most up-regulated genes in *T. aurea*, *H. impetiginosus* and *H. serratifolius* (Fig. 7). In addition to the *AREB* genes, *MYB*, *WRKY*, *NAC*, *ERF* and *NF-Y* are all TF families shown to be involved in the plant response to drought (for a review see [65]) and exhibited many DEG in our study (Fig. 7). There is strong evidence of crosstalks and interaction among these transcription factors when plants are exposed to drought [65, 66]. Overall, the large number and diversity of TFs among down- and up-regulated genes support the important role of these master regulators in the transcriptional responses observed in the *Tabebuia* and *Handroanthus* species.

## Conclusion

To the best of our knowledge, this is the first report of an RNA-Seq study comparing phylogenetically close tree species from seasonally dry tropical forest and savanna ecosystems. Our findings suggest that savanna species are less responsive to drought at the transcriptional level than SDTF species, at least under the water restrictions applied in our study (40% of field capacity). The species with the highest geographic range and widest edaphic-climatic niche, *H. serratifolius*, was the most responsive at the transcriptional level, with the highest number of DEGs. On the other hand, the species with narrower niche, *H. ochraceus,* was practically unresponsive to drought at the transcriptional level. Our findings also show that most of the transcriptional-level responses to drought stress were specific to each of the four analyzed species. This reinforces the complex genetic control of tolerance to drought stress and indicates that habitat and phylogenetic relationships may not be good predictors of possible responses to water deficit. Nevertheless, further studies are needed with more species adapted to contrasting ecosystems, in terms of water restrictions, to draw a more general conclusion.

## Methods

### Experimental design and sampling

We performed RNA-Seq analyses in four species of the Bignoniaceae family. Two are found mainly in savannas (*T. area* and *H. ochraceus*) and two are from seasonally dry forests (*H. impetiginosus* and *H. serrratifolius*). For each species, we collected seeds from three individual trees on and near the Campus of Universidade Federal de Goiás (UFG), in Goiânia, Brazil. The collection was registered in the Brazilian Ministry of the Environment genetic resources system (SisGen code A056151). As such, this collection complies with the national guidelines for research with natural resources (See Ethics Declaration for more details). Species identification was confirmed by comparing each mother tree specimen to exsiccate UFG1304 for *T. aurea*, UFG26994 for *H. impetiginosus*, UFG49125 for *H. ochraceus* and UFG28584 for *H. serratifolius* from the UFG herbarium (Universidade Federal de Goiás, Goiânia, GO).

Seeds were planted in November 2013 in 5 L plastic bags in a nursery (open environment) of Universidade Federal de Goias, in Goiânia-GO, Brazil. Plants grew under a completely randomized block design with 4 species × 2 treatments (drought and wet) × 3 blocks (24 plants). Bags were filled with 4.5 Kg of soil collected in a Cerrado fragment. Physico-chemical analyses indicate that the soil was sandy (56% sand and 38% clay), had pH of 4.8 (in CaCl_2_), low base saturation (36.7%) and aluminum content of 6.9%, which is typical of Cerrado. No fertilization was applied to simulate the Brazilian savanna conditions.

The seedlings were watered every 24 h for 10 months. After this period, on August 2014 at the peak of the dry season, wet treatment plants continued to be watered every 24 h, keeping the soil at field capacity, whereas for the drought treatment plants irrigation was suspended. The pots of drought treated plants were weighed daily to measure the decrease in soil moisture. When the soil of the drought plants reached 40% field capacity, 10 days after treatment, the experiment was considered complete and plants from both treatments were collected. After this period of water suppression, some plants showed visible signs of water stress, such as wilting and senescing leaves (Additional File 2: Fig. S4). The shoots (stem and leaves) and roots of all the plants were weighed separately, to measure above and below-ground biomass. The shoots were collected for RNA extraction and sequencing, while the roots were dried at 120 °C and weighed until constant mass. Two *H. ochraceus* plants died during the experiment (one from the wet and one from the drought stress treatment). An additional wet sample of *H. ochraceus* was lost during quality control of the sequences. These three *H. ochraceus* samples were excluded from analyses.

### RNA sequencing and read processing

Total RNA from shoots of the 21 remaining plants was extracted using a Qiagen RNeasy Plant Mini kit (Qiagen, DK). Libraries were prepared using the TruSeq Stranded mRNA Sample Prep kit (Illumina, CA, USA) and sequenced at BGI Genomics in Hong Kong, China. Paired-end sequencing, with 2 × 100 nt, was performed in three lanes of an Illumina HiSeq 2000 instrument (Illumina, CA, USA). In each lane, samples from each block of the experiment were barcoded and sequenced.

RNA-Seq reads were evaluated with FastQC (www.bioinformatics.babraham.ac.uk/projects/fastqc/) for quality control (see analysis pipeline in Additional File 2: Fig. S5). Next, Trimmomatic [67] removed low quality sequences and the Illumina adapters. The following parameters were used in Trimmomatic: ILLUMINACLIP:TruSeq3-PE-2.fa:2:15:7 SLIDINGWINDOW:4:5 MINLEN:25. The reads were mapped to the reference genome of *H. impetiginosus*, which has ~ 557 Mbp and 31,688 genes [40], using STAR software [68]. The following parameters were used in STAR: --outFilterMismatchNoverLmax 0.5 --outFilterMatchNmin 16 --outFilterScoreMinOverLread 0 --outFilterMatchNminOverLread 0 --alignMatesGapMax 1000 --alignIntronMax 10,000 --outFilterType BySJout --outFilterMultimapNmax 20 --outSAMtype BAM Unsorted --alignSJoverhangMin 8. Alignment quality was analyzed using SAMStat [69].

### Analyses of differential expression

The HTSeq-count script [70] was used to count the number of reads in each sample that mapped to only one gene annotated in the reference genome (Additional File 2: Fig. S5). For each sample, the number of reads uniquely mapped to each gene was used as an estimate of gene expression. The gene counting vector for each sample was combined into a matrix, in R software, with genes as rows and the 21 samples (species × treatments × replicates) as columns. Genes with a total count of less than 50 across the 21 samples were excluded from further analyses. The statistical power is lower for these genes and they increase the need for multiple test corrections with false discovery rate (FDR) [71].

In order to identify differentially expressed genes (DEG), the counting matrix was analyzed using the Bioconductor packages DESeq2 [72] and edgeR [73]. Both programs apply statistical methods based on negative binomial distribution to normalize against differences in library size, preventing bias from the relative sample size of the transcriptomes (for a review, see [74]). The Relative Log Expression (RLE) method and Trimmed Mean of M-values (TMM) were used for DESeq2 and edgeR, respectively.

We obtained differentially expressed genes (DEGs) between treatments (wet and drought) for each species, using the threshold of FDR smaller than 0.05. In order to minimize false negatives, only the DESeq2 results were used for the follow-up functional analyses. A Venn diagram implemented in the online tool Venny 2.1 [75] was used to identify conserved expressed genes (CEG), namely DEG with the same direction of expression among pairwise species (e.g. genes up-regulated in both species). The remaining genes were further classified as exclusive, when differentially regulated in a single species, or diverged expressed genes (DiEG) when the direction of expression was opposite among pairwise species (e.g. up-regulated in one and down-regulated in the other). In other words, CEG was defined as orthologs with the same pattern of expression in different species, i.e. up- or down-regulated in two or more species; DiEG are orthologs with a different pattern of expression, i.e. up-regulated in one species and down-regulated in the other. The list of DEG, classified as CEG and DiEG, is provided as Additional File 1 (Table S6).

### Functional annotation and transcription factor analysis

Functional annotation of all DEGs was performed using Gene Ontology (GO, [76]), KEGG Orthology (KO, [77]), Eukaryotic Orthologous Groups (KOG, [78]), PANTHER [79] and PFAM [80]*.* In addition, UniProt [81] protein annotation and the Blastp [82] best hit for *Arabidopsis thaliana* is also provided, along with its gene definition. This annotation is provided as supplementary material (Additional File 1: Table S6) and significantly improves the functional categorization of genes from the *H. impetiginosus* genome [40]. Functional enrichment analysis was performed using Fisher’s exact test with FDR < 0.05, in the AgriGO 2.0 online tool [83]. GO term sharing among species was analyzed with Venn diagrams using Venny software [75]. The metabolic pathways of DEGs were visualized using iPath3 [84] and the KO terms to identify pathways potentially affected by drought in each species.

Putative transcription factors among DEGs were identified using the prediction tool provided by the Plant Transcription Factor Database 4.0 (PlantTFDB - http://planttfdb.gao-lab.org/) [85]. Nucleotide sequences from DEGs were separated according to their expression level. Thus, each species had two input files: one for up-regulated and one for down-regulated genes. Up- and down-regulated transcription factors were identified and classified in terms of PlantTFDB gene families (e.g. MYB, NAC, WRKY, etc.) for each species.

## Supplementary Information

**Additional file 1 **Supplementary Tables with summary of sequence data analysis and results of functional annotation and enrichment analysis, acronyms and Transcription Factor Families among differentially expressed genes (DEGs), including Tables S1 to S7. **Table S1** Summary of sequence data for each library before and after filtering. % loss is the percentage of reads removed after filtering; **Table S2** Alignment of RNA-seq reads of each library in the reference genome of *Handroanthus impetiginosus*, using the software STAR. % mapping is the percentage of reads mapped to one contig of the reference genome; % multiple is the percentage of reads mapped to more than one contig; % non-alignment is the percentage of reads not aligned to the reference genome; % MAPQ > = 30 is the percentage of reads with mapping alignment > = 30; **Table S3** Functional enrichment of GO terms for up- and down-regulated genes in *Tabebuia aurea*, based on the Fisher exact test with a false discord rate (FDR) < 0.05. Ontology is the class of gene function; F, molecular function; P, biological process; C, cellular component; Number in BG/Ref, number of GO terms in the reference genome; **Table S4** Functional enrichment of GO terms for up and down-regulated genes in *Handroanthus impetiginosus*, based on the Fisher exact test with a false discord rate (FDR) < 0.05. Ontology is the class of gene function; F, molecular function; P, biological process; C, cellular component; Number in BG/Ref, number of GO terms in the reference genome; **Table S5** Functional enrichment of GO terms for up and down-regulated genes in *Handroanthus serratifolius*, based on the Fisher exact test with a false discord rate (FDR) < 0.05. Ontology is the class of gene function; F, molecular function; P, biological process; C, cellular component; Number in BG/Ref, number of GO terms in the reference genome; **Table S6** Functional anotation of *Handroanthus impetiginosus* genes with differential expression represented by log2-fold-change, in *T. aurea*, *H. ochraceus*, *H. impetiginosus* and *H. serratifolius*. The table shows shared and exclusive expressed genes among species, based on the reference genome annotation. Gene_name: gene name of *H. impetiginosus* genes; Acession: Acession identifier to *H. impetiginosus* genes on NCBI; Protein_product: Protein identifier to *H. impetiginosus* products on NCBI; Uniprot_name: Uniprot identifier to *H. impetiginosus* genes; Best_hit: Blast best hit result against *Arabidopsis thaliana* proteins (v. Araport11); Description: description of *A. thaliana* genes; GO_Term: gene ontology term; EC_number: enzyme comission number of enzyme; KO_identifier: KEGG orthology identifier; KOG: eukaryotic orthologous group; PANTHER: protein alnalysis through evolutionary relationships; PFAM: protein families; log2FC: log2-fold-change; padj: adjusted *p*-value with false discovery rate (FDR); CEG: conserved expressed genes; DiEG: divergent expressed genes; **Table S7** Description of the acronyms of Transcription Factors Family among differentially expressed genes (DEGs) in *H. impetiginosus, H. ochraceus, H. serratifolius* and *T. aurea. (XLSX 5179 kb)*

**Additional file 2 **Additional figures: Heatmap with differentially expressed genes (Fig. S1), biomass information (Fig. S2) and pipeline (Fig. S3). **Fig. S1.** Heatmap of the differentially expressed genes (DEGs) with false ratio discovery (FDR) < 0.05 for *Tabebuia aurea* **a**, *Handroanthus ochraceus* **b**, *H. impetiginosus* **c** and *H. serratifolius* **d**. Red represents down-regulated genes and green represents up-regulated genes under drought. The cluster trees show the relationship among replicates at rows and among DEGs at columns. **Fig. S2** Jaccard distance among samples using Gene Ontology (GO) enriched categories as binary markers. GO categories with enrichment in a sample assumed value of “1” and those with lack of enrichment had value “0” in each sample. a) Dendrogram representing the Jaccard distance matrix, with cophenetic correlation of 0.95 (p-value = 0.0056 by Mantel test). b) Principal component analyses of the Jaccard distance matrix, with cophenetic correlation of 0.82 (p-value < 0.0001 by Mantel test). **Fig. S3.** Wet root biomass, dry root biomass, wet shoot biomass and biomass allocation for each species in the irrigated and drought plant groups. Biomass allocation is unitless as it is the ratio between shoot and root biomass. Panels are on different scales. Variation is depicted by standard error. Different plants were submitted to each treatment (drought vs. irrigated). The dry biomass was only obtained for roots because the shoots were used for RNA extraction. **Fig. S4** Pictures of plants from seasonally dry tropical forest (*H. serratifolius* and *H. impetiginosus*, on top) and savanna (*H. ochraceus* and *T. aurea*, on bottom) submitted to drought and irrigated (control) treatments. Drought caused wilting and senescence in *H. serratifolius* and wilting in *H. impetiginosus*, while savanna species did not show any visible symptom. **Fig. S5.** Bioinformatics pipeline for RNA-seq processing to identify differentially expressed genes (DEGs) and functionally annotate them. The raw FASTQ files were submitted to a quality control procedure via FASTQC package to identify low-quality reads. Adaptors and contaminated reads were removed using Trimmommatic. STAR was used for mapping the reads to the reference genome, and alignment quality was checked using SAMStat. HTSeq counts the number of reads aligned in each gene, aiding the expression data used for subsequent differential expression analysis with edgeR and DESeq2. Up-and down-regulated genes obtained from DESeq2 were summarized using Venn diagrams in Venny to show conserved (CEGs) and diverged (DiEG) differentially expressed genes. Functional annotation of DEGs was performed using Gene Ontology (GO) and KEGG database. KEGG identifiers were used in iPath3 to plot differentially expressed genes in the main metabolic pathways. A Fisher Exact test was carried out to identify enriched GO terms using agriGO and then a Venn diagram was obtained to identify shared and exclusive GO enriched terms. Transcription factors annotation among differentially expressed genes was also performed using PlantTFDB database.

## Data Availability

All functional annotation data generated during this study are included as supplementary material files (Table S6). RNA-seq data is available in the SRA database (BioProject accession PRJNA608000) under the link https://www.ncbi.nlm.nih.gov/bioproject/PRJNA608000

## Abbreviations

ABA: Abscisic Acid
*ABRE*: ABA-responsive element
ATP: Adenosine triphosphate
BES1: BRI1-EMS-Suppressor 1
*bHLH*: Basic helix-loop-helix
bZIP: Basic Leucine Zipper
*C2H2*: Cys2His2 zinc finger
*C3H*: Cys3His zinc finger
*CEG*: Conserved Expressed Genes
DiEG: diverged expressed genes
DEG: Differentially Expressed Genes
*DOF*: DNA-binding with one zinc finger
*ERF*: Ethylene-Responsive factor
*FAR1*: FAR-red-impaired response1
FDR: False Discovery Rate
*G2-like*: Golden2-like
GO: Gene Ontology
*HD-ZIP*: Homeodomain-leucine Zipper
*HSF*: Heat Shock Factor
JA: Jasmonic Acid
KO: KEGG Orthology
KOG: EuKaryotic Orthologous Groups
MAPQ: mapping quality
*M-type MADS*: M-type MCM1, Agamous, Deficiens and Serum response factor
*MYB*: Myeloblastosis
*NAC*: NAM, ATAF1/2, and CUC2 transcription factors
*NF-YA*: Nuclear transcription Factor Y subunit Alpha
*NF-YC*: Nuclear transcription Factor Y subunit Gamma
NGS: Next-Generation Sequencing
PANTHER: Protein ANalysis THrough Evolutionary Relationships
PFAM: Protein Families
RLE: Relative Log Expression
RNA-seq: RNA sequencing
ROS: Reactive Oxygen Species
rRNA: Ribosomal RNA
SDTF: Seasonally Dry Tropical Forest
TF: Transcription Factor
TMM: Trimmed Mean of M-values
