# Supplementary material for: RNA-Seq reveals different responses to drought in Neotropical trees from savannas and seasonally dry forests
Source: BMC Plant Biol. 2021 Oct 12;21:463. doi: 10.1186/s12870-021-03244-7 (PMC8507309; doi:10.1186/s12870-021-03244-7)
Supplement: Supplementary file 2 — Additional file 2 Additional figures: Heatmap with differentially expressed genes (Fig. S1), biomass information (Fig. S2) and pipeline (Fig. S3). Fig. S1. Heatmap of the differentially expressed genes (DEGs) with false ratio discovery (FDR) < 0.05 for Tabebuia aurea a, Handroanthus ochraceus b, H. impetiginosus c and H. serratifolius d. Red represents down-regulated genes and green represents up-regulated genes under drought. The cluster trees show the relationship among replicates at rows and among DEGs at columns. Fig. S2 Jaccard distance among samples using Gene Ontology (GO) enriched categories as binary markers. GO categories with enrichment in a sample assumed value of “1” and those with lack of enrichment had value “0” in each sample. a) Dendrogram representing the Jaccard distance matrix, with cophenetic correlation of 0.95 (p-value = 0.0056 by Mantel test). b) Principal component analyses of the Jaccard distance matrix, with cophenetic correlation of 0.82 (p-value < 0.0001 by Mantel test). Fig. S3. Wet root biomass, dry root biomass, wet shoot biomass and biomass allocation for each species in the irrigated and drought plant groups. Biomass allocation is unitless as it is the ratio between shoot and root biomass. Panels are on different scales. Variation is depicted by standard error. Different plants were submitted to each treatment (drought vs. irrigated). The dry biomass was only obtained for roots because the shoots were used for RNA extraction. Fig. S4 Pictures of plants from seasonally dry tropical forest (H. serratifolius and H. impetiginosus, on top) and savanna (H. ochraceus and T. aurea, on bottom) submitted to drought and irrigated (control) treatments. Drought caused wilting and senescence in H. serratifolius and wilting in H. impetiginosus, while savanna species did not show any visible symptom. Fig. S5. Bioinformatics pipeline for RNA-seq processing to identify differentially expressed genes (DEGs) and functionally annotate them. The raw FAST [file 12870_2021_3244_MOESM2_ESM.docx]

**RNA-Seq reveals different responses to drought in Neotropical trees from savanna and seasonally dry forests**

**Mariane B. Sobreiro^1^, Rosane G. Collevatti^1^, Yuri L. A. dos Santos^2^, Ludmila F. Bandeira^2^, Francis J. F. Lopes^3^, Evandro Novaes^4*^**

^1^Laboratório de Genética & Biodiversidade, Instituto de Ciências Biológicas, Universidade Federal de Goiás, Goiânia, GO, Brazil, 74690-900;

^2^Laboratório de Genética e Genômica de Plantas, Escola de Agronomia, Universidade Federal de Goiás, Goiânia, GO, Brazil, 74690-900;

^3^Laboratório de Fisiologia Vegetal, Instituto de Ciências Biológicas, Universidade Federal de Goiás, Goiânia, GO, Brazil, 74690-900;

^4^Laboratório de Genética Molecular, Departamento de Biologia, Universidade Federal de Lavras, MG, Brazil, 37200-900.

*** Author for correspondence:** Evandro Novaes, Departamento de Biologia, Universidade Federal de Lavras, 37200-900, Lavras, MG, Brazil. E-mail: [evandro.novaes@ufla.br](mailto:evandro.novaes@ufla.br)*.* Phone: +55 35 3829-1357

**Additional File 2.** Additional figures: Heatmap with differentially expressed genes (Figure S1), Jaccard distance among samples with GO enrichment (Figure S2), biomass information (Figure S3) and pipeline (Figure S4).


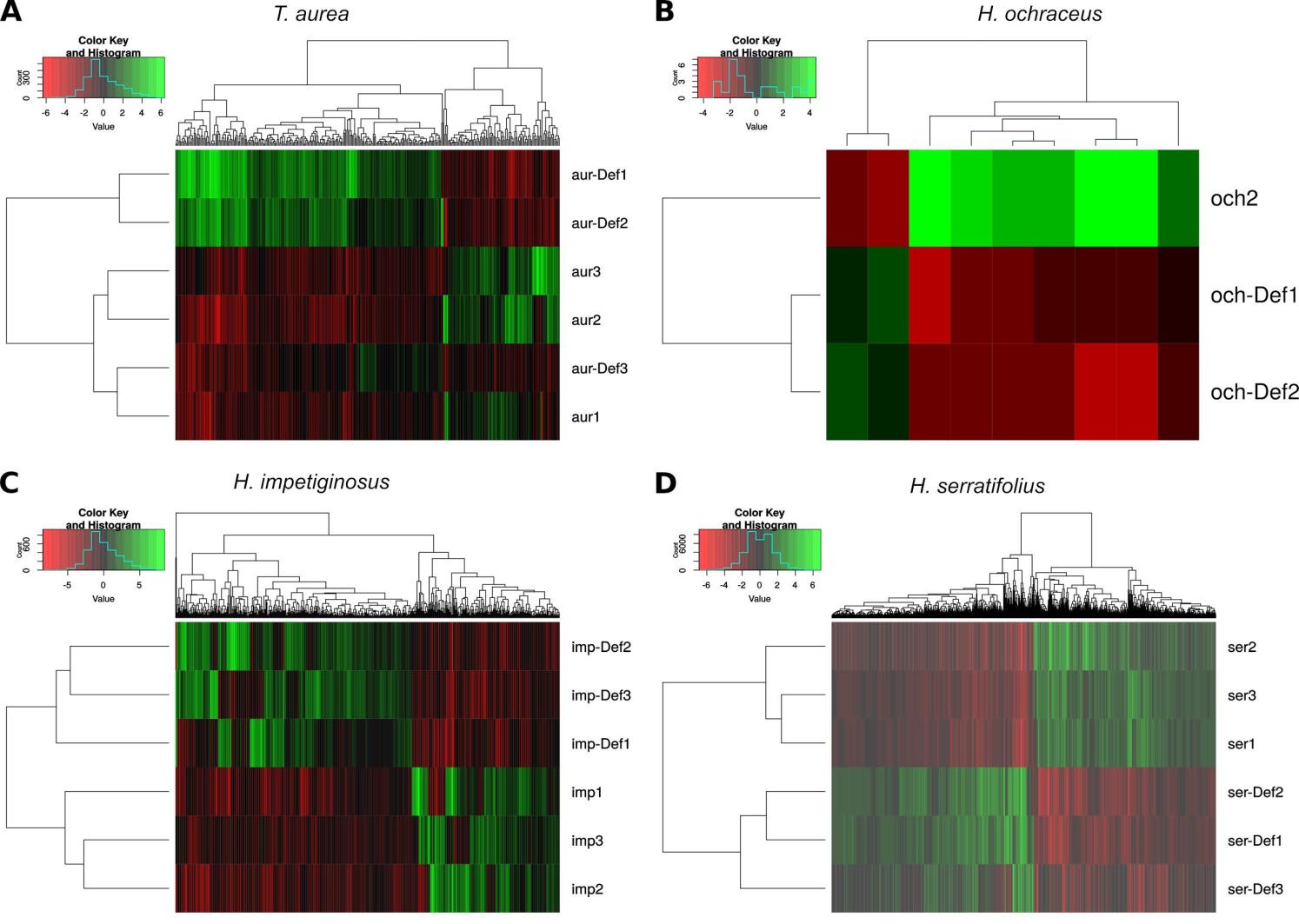


**Figure S1** Heatmap of the differentially expressed genes (DEGs) with false ratio discovery (FDR) < 0.05 for *Tabebuia aurea* (A), *Handroanthus ochraceus* (B), *H. impetiginosus* (C) and *H. serratifolius* (D). Red represents down-regulated genes and green represents up-regulated genes under drought. The cluster trees show the relationship among replicates at rows and among DEGs at columns


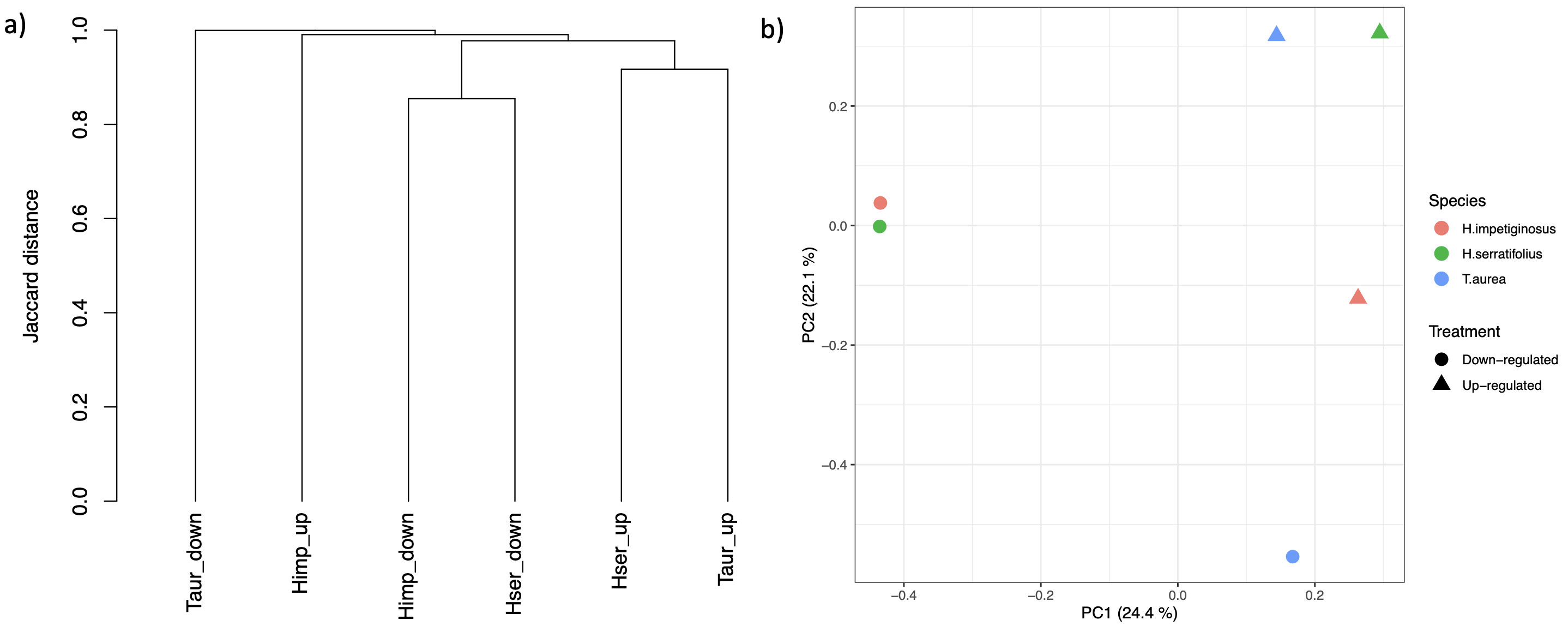


**Figure S2** Jaccard distance among samples using Gene Ontology (GO) enriched categories as binary markers. GO categories with enrichment in a sample assumed value of “1” and those with lack of enrichment had value “0” in each sample. a) Dendrogram representing the Jaccard distance matrix, with cophenetic correlation of 0.95 (p-value = 0.0056 by Mantel test). b) Principal component analyses of the Jaccard distance matrix, with cophenetic correlation of 0.82 (p-value < 0.0001 by Mantel test).


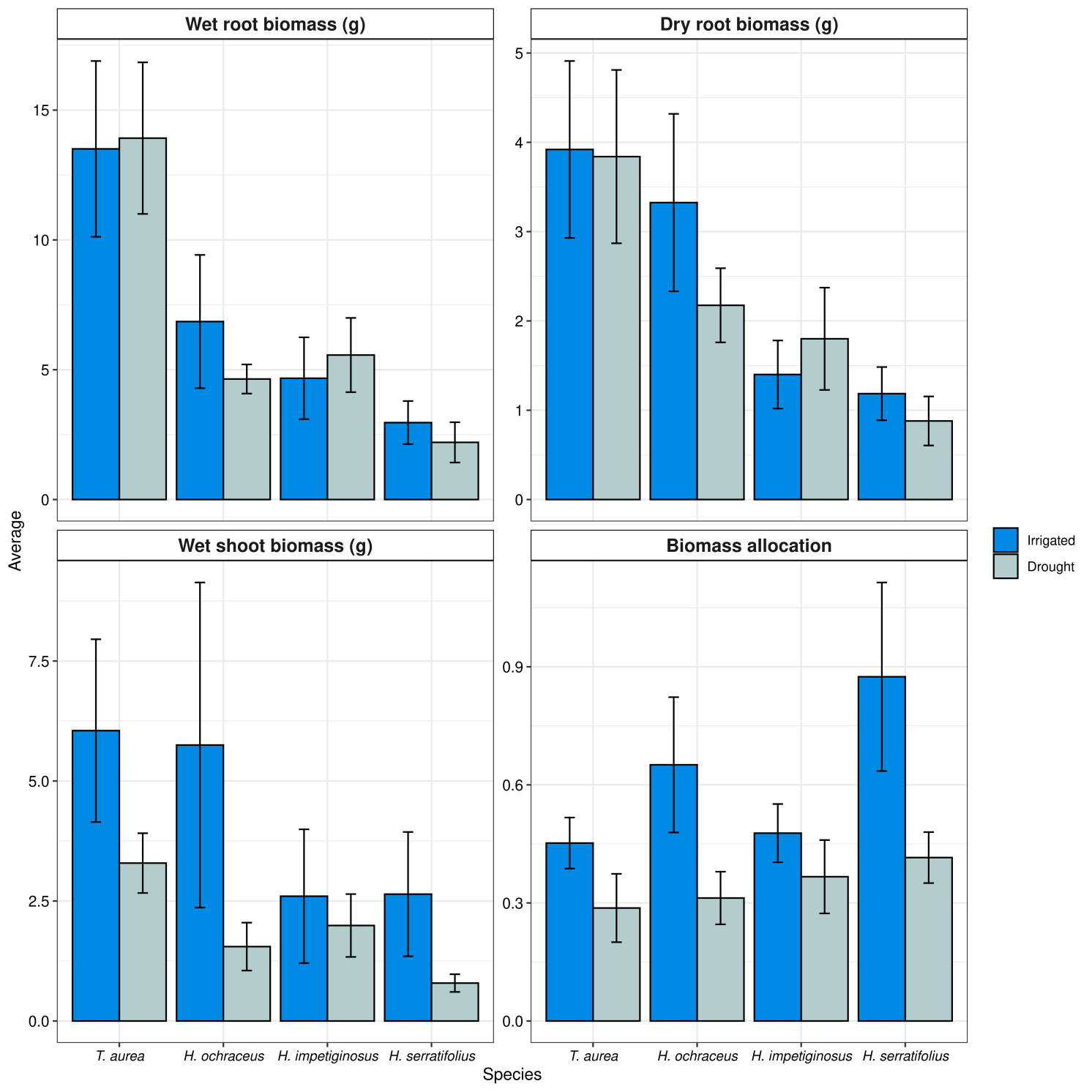


**Figure S3** Wet root biomass, dry root biomass, wet shoot biomass and biomass allocation for each species in the irrigated and drought plant groups. Biomass allocation is unitless as it is the ratio between shoot and root biomass. Panels are on different scales. Variation is depicted by standard error. Different plants were submitted to each treatment (drought vs. irrigated). The dry biomass was only obtained for roots because the shoots were used for RNA extraction.


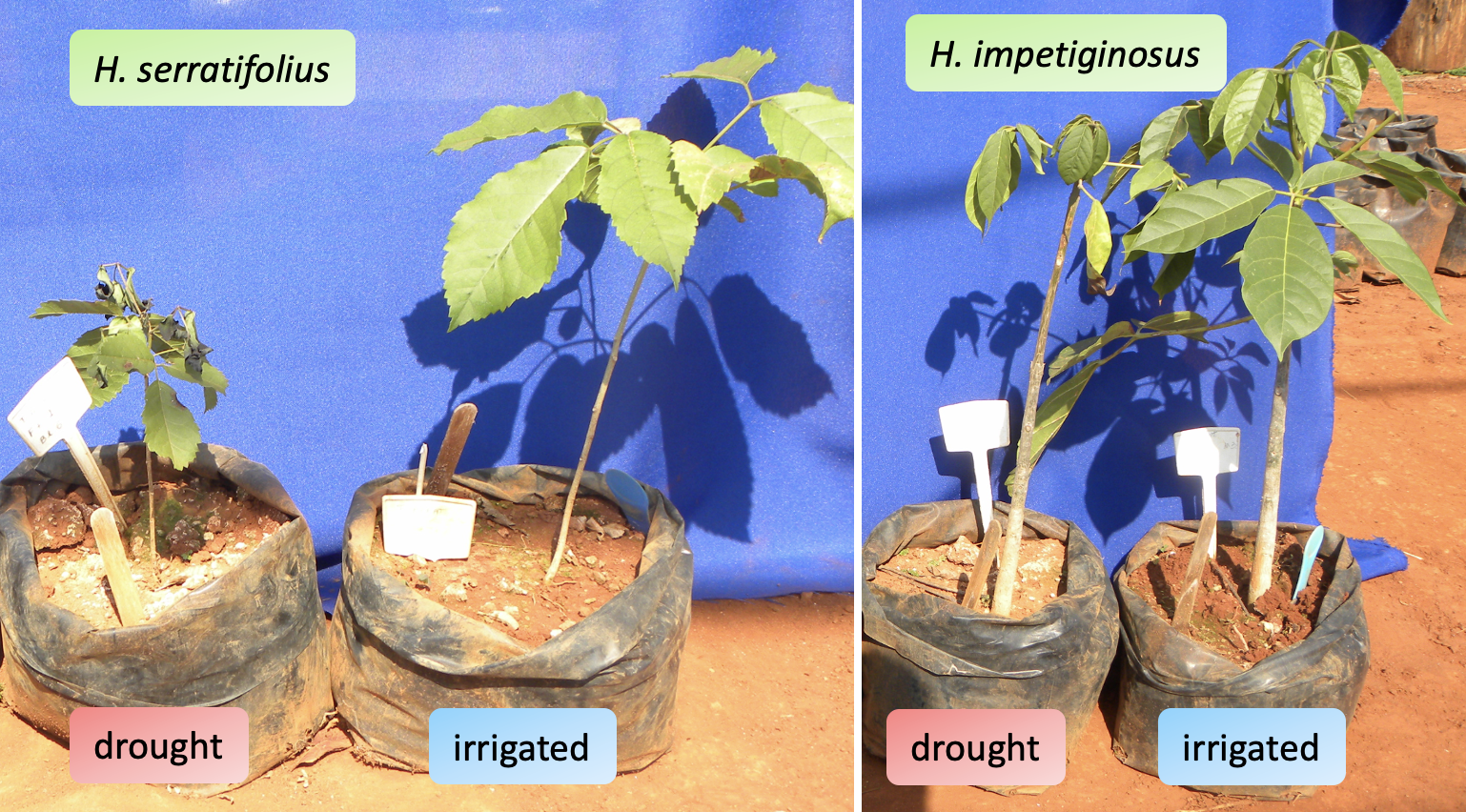


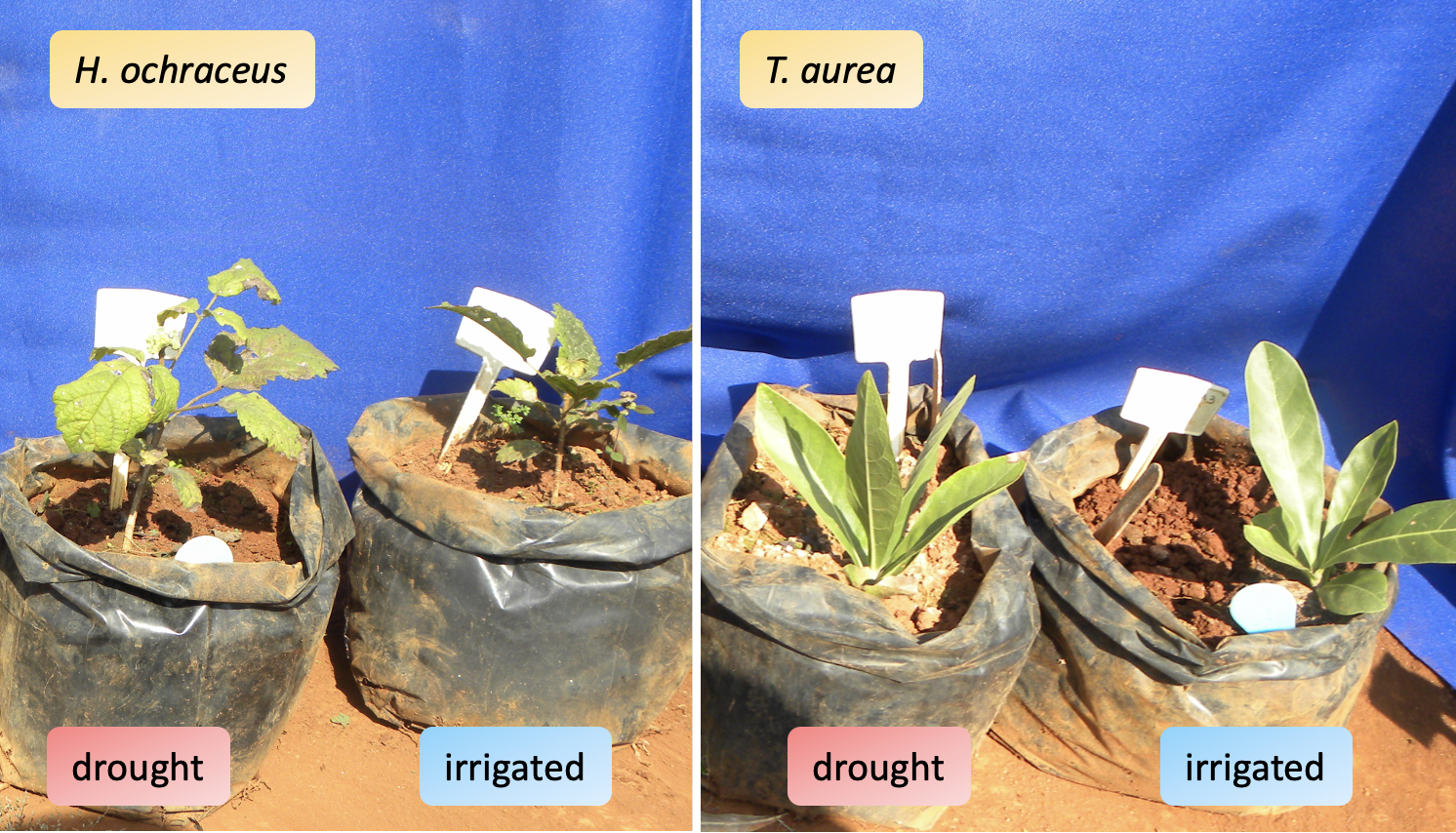


**Figure S4** Pictures of plants from seasonally dry tropical forest (*H. serratifolius* and *H. impetiginosus*, on top) and savanna (*H. ochraceus* and *T. aurea*, on bottom) submitted to drought and irrigated (control) treatments. Drought caused wilting and senescence in *H. serratifolius* and wilting in *H. impetiginosus*, while savanna species did not show any visible symptom.


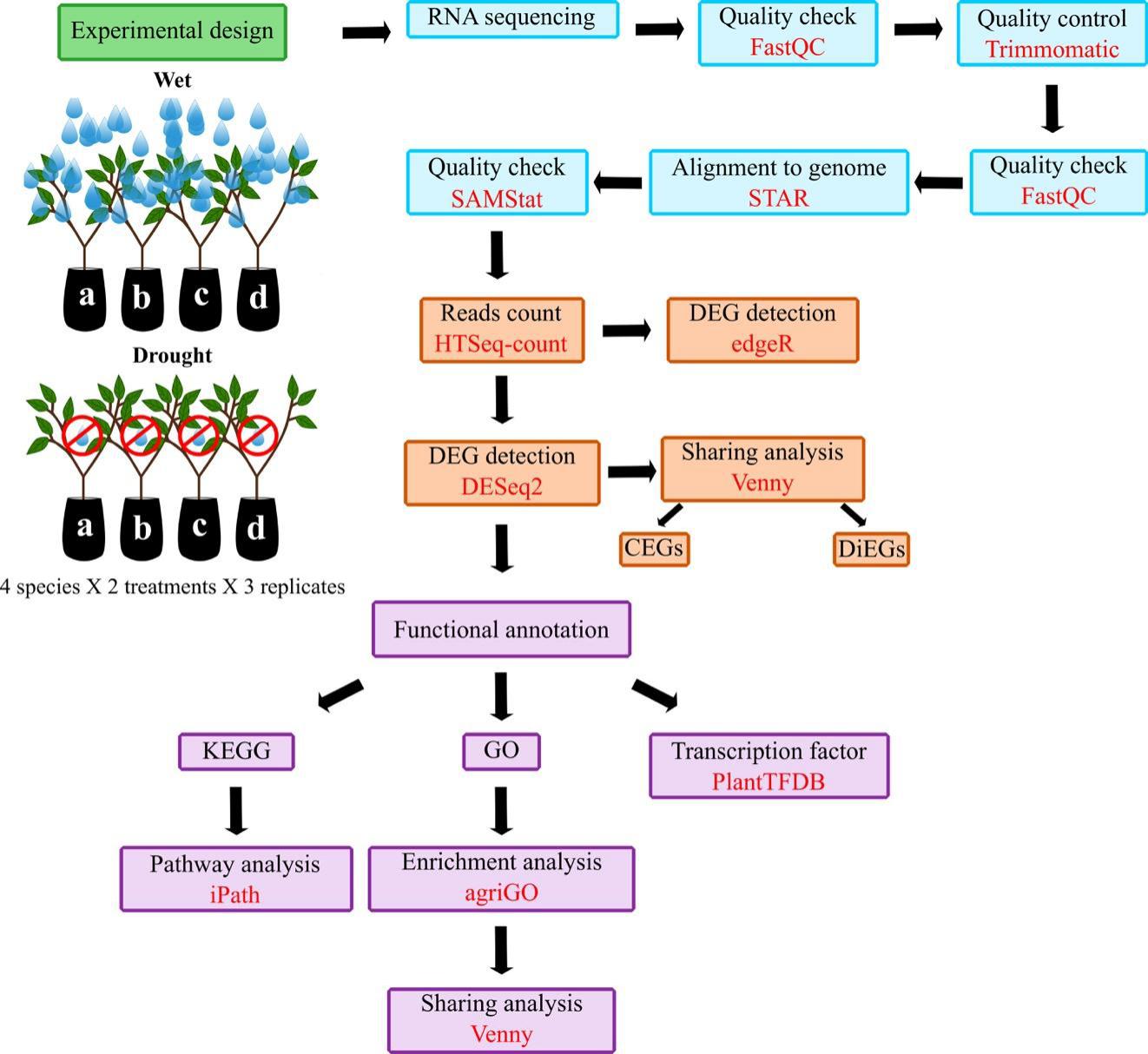


**Figure S5** Bioinformatics pipeline for RNA-seq processing to identify differentially expressed genes (DEGs) and functionally annotate them. The raw FASTQ files were submitted to a quality control procedure via FASTQC package to identify low-quality reads. Adaptors and contaminated reads were removed using Trimmommatic. STAR was used for mapping the reads to the reference genome, and alignment quality was checked using SAMStat. HTSeq counts the number of reads aligned in each gene, aiding the expression data used for subsequent differential expression analysis with edgeR and DESeq2. Up-and down-regulated genes obtained from DESeq2 were summarized using Venn diagrams in Venny to show conserved (CEGs) and diverged (DiEG) differentially expressed genes. Functional annotation of DEGs was performed using Gene Ontology (GO) and KEGG database. KEGG identifiers were used in iPath3 to plot differentially expressed genes in the main metabolic pathways. A Fisher Exact test was carried out to identify enriched GO terms using agriGO and then a Venn diagram was obtained to identify shared and exclusive GO enriched terms. Transcription factors annotation among differentially expressed genes was also performed using PlantTFDB database
